# A regulatory loop involving miR-29c and Sp1 elevates the TGF-β1 mediated epithelial-to-mesenchymal transition in lung cancer

**DOI:** 10.18632/oncotarget.13137

**Published:** 2016-11-05

**Authors:** Hai-wei Zhang, En-wen Wang, Li-xian Li, Shou-hui Yi, Lu-chun Li, Fa-liang Xu, Dong-lin Wang, Yong-zhong Wu, Wei-qi Nian

**Affiliations:** ^1^ Key Laboratory of Oncology, Chongqing Cancer Institute, Chongqing Cancer Hospital, Chongqing Cancer Center, Chongqing, China; ^2^ Chongqing Key Laboratory of Translational Research for Cancer Metastasis and Individualized Treatment, Chongqing Cancer Institute, Chongqing, China; ^3^ Department of Oncology, Chongqing Cancer Institute, Chongqing, China; ^4^ Center of Breast Cancer, Chongqing Cancer Institute, Chongqing, China; ^5^ Department of Radiotherapy, Chongqing Cancer Institute, Chongqing, China

**Keywords:** miR-29c, metastasis, TGF-β1, sp1, EMT

## Abstract

Specificity protein1 (Sp1) is required for TGF-β-induced epithelial-to-mesenchymal transition (EMT) which has been demonstrated to aggravate the progression of cancer including lung cancer. microRNA-29c (miR-29c) is identified to inhibit EMT, but the correlation between miR-29c and Sp1 in human lung cancer remain incompletely clarified. Here, we confirmed decreased expression of miR-29c and enhanced expression of Sp1 in lung cancer tissues (*n* = 20) and found that Sp1 could be targeted and inhibited by miR-29c. Besides, the expression of miR-29c was down-regulated in high-metastatic lung cancer cell lines and TGF-β1-treated cells. The inhibition of miR-29c or overexpression of Sp1 in 95C and A549 cells dramatically enhanced the cell migration and invasion, and also induced the decrease in the expression of epithelial markers, e.g. thyroid transcription factor 1 (TTF-1) and E-cadherin, together with an increase in mesenchymal markers including vimentin, α-smooth muscle actin (α-SMA), which could be restored by overexpression of miR-29c mimics during the TGF-β-induced EMT. Moreover, dual-luciferase reporter assay was performed and the results indicated that miR-29c/Sp1 could form an auto-regulatory loop with TGF-β1, which impaired TGFB1 transcription. Furthermore, miR-29c overexpression could abrogate the tumor progression and inhibit the Sp1/TGF-β expressions *in vivo*, indicating that miR-29c could be a tumor suppressor and repress the Sp1/TGF-β axis-induced EMT in lung cancer.

## INTRODUCTION

Lung cancer is one of the most common cancers worldwide and a leading cause of cancer-related mortality in men and women [1]. Non–small cell lung cancer (NSCLC) and small cell carcinoma lung cancer (SCLC) is two main groups of lung cancer. The 5-year survival rates of NSCLC patients diagnosed at localized stage was approximately 50% [2, 3]. Amounts of evidences proved the epithelial-to-mesenchymal transition (EMT) to be a key step for carcinogenesis which enables tumor cells to become invasive, survive in the circulation, extravasate, and ultimately establish the secondary tumor sites [4, 5]. Currently, transforming growth factor beta (TGF-β) is the prominent signaling molecules to induce EMT, which is characterized by down-regulation of epithelial markers including E-cadherin, keratins and thyroid transcription factor 1 (TTF-1), up-regulation of mesenchymal markers including such as vimentin and N-cadherin.

Specificity protein1 (Sp1) is one of the most well characterized transcriptional factors and belongs to the Sp/Kruppel like factor (KLF) family [6]. Clinical evidences showed that Sp1 is over-expressed in a number of cancers and have been show to correlate with cancer cell migration and metastasis via the regulation of EMT in various cancers, such as gastric [7], pancreatic [8], breast cancers [9] and hepatocellular carcinoma [10]. In human glioma, overexpression of Sp1 was demonstrated to increase the invasiveness of glioma cells via increased activity and expression of MMP-2, and was positively correlated with WHO grades of glioma [11]. Choi et al. had reported that Sp1 binding site in the Slug promoter was responsible for TGF-β-induced Slug expression and the repression of E-cadherin in patients with cataracts [12]. Nevertheless, the mechanism of Sp1 in the regulation of TGF-β-induced EMT in lung cancer needs to be further investigated.

Mature miRNAs are short, single-stranded, endogenous and non-coding RNAs consisting of about 22 nucleotides, which regulate genes at the post-transcriptional level during the translation process [13]. In human colorectal carcinoma (CRC), zhang et al. had found that miR-29c could inhibit EMT and impair Wnt/β-catenin signaling pathway. In addition, miR-29c impaired the CRC cells migration and invasion abilities *in vitro* and cancer metastasis *in vivo* by directly targeting guanine nucleotide binding protein alpha13 (GNA13) and protein tyrosine phosphatase type IVA (PTP4A) [14]. Although miR-29c was reported to directly target the 3′ UTR of Sp1 to repress its expression and regulated type I collagen production under TGF-β1-stimulated kidney fibrosis [15], the relationship between Sp1 and miR-29c in TGF-β-induced EMT during the development of lung cancer was incompletely known.

In this study, we demonstrated that Sp1 could be targeted by miR-29c in translational level, and TGF-β1 inhibit the expression of miR-29c and up-regulate expression of Sp1 *in vitro*. Inhibition of miR-29c or Sp1 overexpression dramatically enhanced the cell migration and invasion via the regulation of TGF-β-induced EMT. Moreover, miR-29c/Sp1 could form an auto-regulatory loop with TGF-β1, which impaired TGFB1 transcription. These findings were confirmed *in vivo* and the mice treated with miR-29c mimics showed impaired tumor progression.

## RESULTS

### MiR-29c targets Sp1 and is down-regulated in lung cancer tissues and high-metastatic lung cancer cell lines

**Figure 1:**
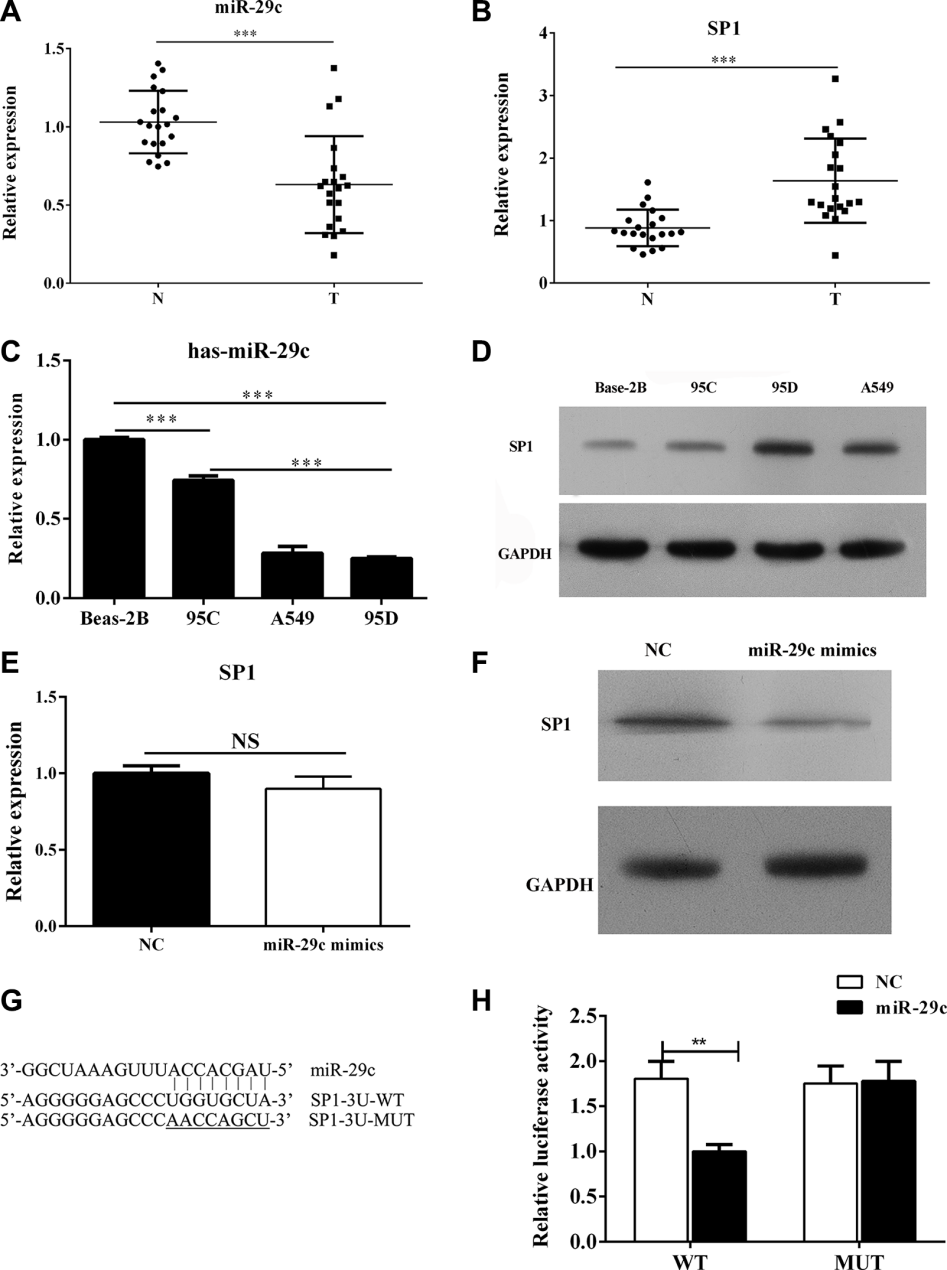
MiR-29c targets Sp1 and is down-regulated in high-metastatic lung cancer cell lines (**A**–**B**) The level of miR-29c and Sp1 in lung cancer tissues and (**C**–**D**) cell lines including BEAS-2B, the paired low-metastatic 95C and high-metastatic 95D and A549 were determined by Q-PCR and Western blotting. 95C cell line was transfected with miR-29c mimics or negative control. (**E**–**F**) the mRNA and protein level of Sp1 were determined. (**G**–**H**) The luciferase reporter was performed to confirm the direct target sites. **p < 0.01, ***p < 0.001, data represent the means ± s.d.

To investigate the role of miR-29c in lung cancer metastasis, lung cancer tissues and corresponding non-neoplastic tissues (*n* = 20) were collected. The results from Q-PCR indicated decreased mRNA expression of miR-29c and enhanced expression of Sp1 in lung cancer tissues (Figure 1A and 1B). Besides, three cell lines were collected in this study: lung/brunch normal epithelial cell line BEAS-2B, the paired low-metastatic 95C and high-metastatic 95D and A549 lung cancer cell lines. The Q-PCR results had shown that the expression of miR-29c was lower in lung cancer cell lines 95C, 95D and A549 than that in the normal human epithelial cell line BEAS-2B (Figure 1C). Interestingly, we also found the expression of miR-29C is remarkably lower in the paired high-metastatic lung cancer cell line 95D when compared to the low-metastatic lung cancer cell line 95C. The transcriptional factors Sp1 was reported to be the target of miR-29c in kidney tubular epithelial cells and we found that the expression of Sp1 was enhanced in lung cancer cell lines 95C, 95D and A549 than that in normal human epithelial cell line BEAS-2B. Meanwhile, we noted that the level of Sp1 was higher in the high-metastatic lung cancer cell line 95D (Figure 1D).

We next verified the relationship between miR-29c and Sp1 in lung cancer. MiRWalk (http://www.umm.uni-heidelberg.de/apps/zmf/mirwalk/) showed that the 3′-UTR of Sp1 mRNA existed binding sites of miR-29c. To conform the directly target relationship, ectopically expressing miR-29c mimics in 95C cell line could significantly reduce the protein levels of Sp1, although the mRNA level of Sp1 was unchanged (Figure 1E and 1F). Furthermore, we conducted a luciferase reporter vector with full-length 3′-UTR (wild-type or mutant) of Sp1 mRNA in 95C cell line to perform the luciferase reporter assays and the results showed that miR-29c mimics could significantly impair the luciferase activity of wild-type Sp1-3′UTR, but not the mutant Sp1-3’UTR (Figure 1G and 1H). These findings implied that miR-29c could directly target Sp1 and the down-regulation of miR-29c might participate in the tumor metastasis.

### Inhibition of miR-29c significantly elevates the migration and invasion of 95C cells

**Figure 2:**
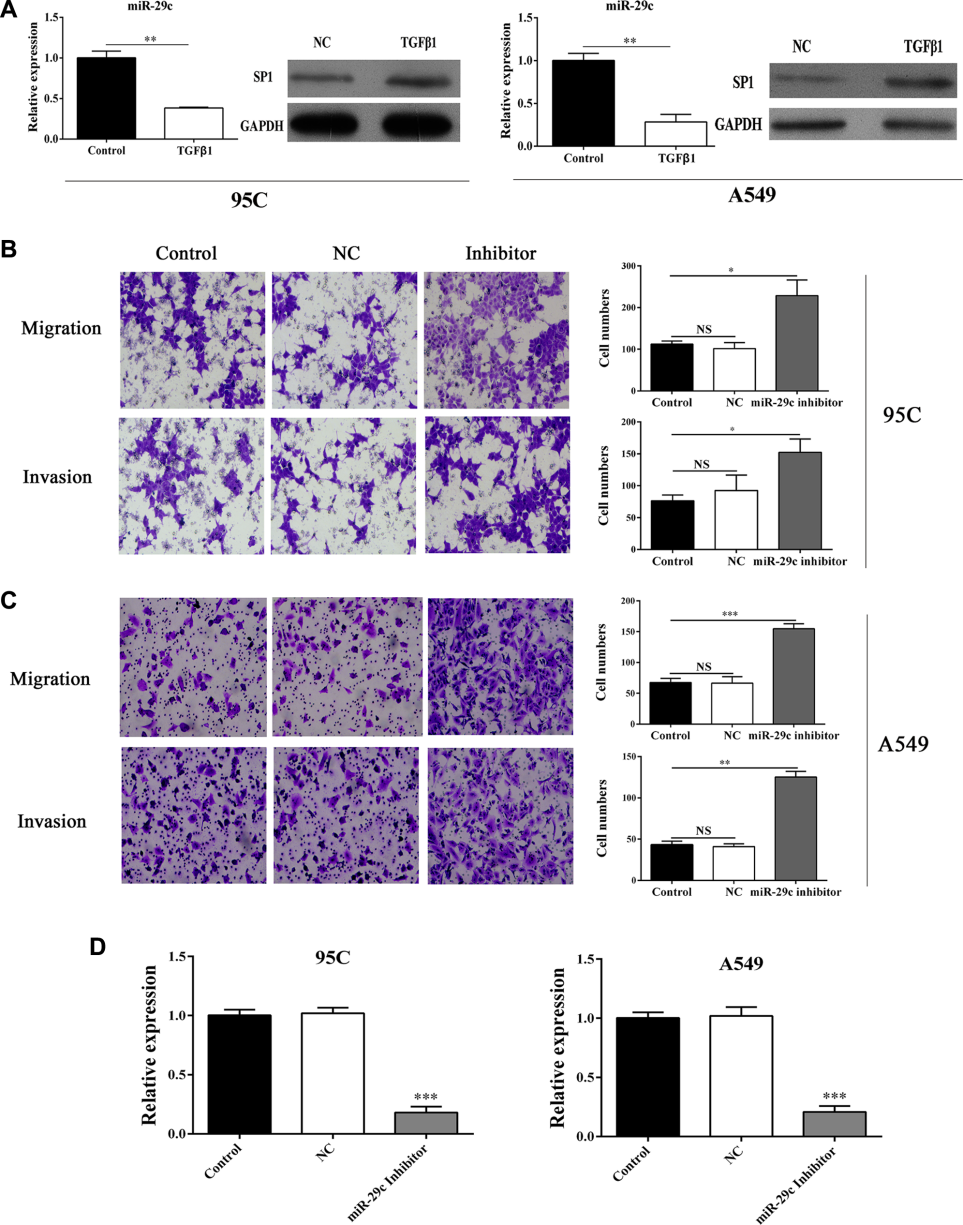
Inhibition of miR-29c significantly elevates the migration and invasion (**A**) The expression of mir-29c and Sp1 in TGF-β1-treated cells. Transwell assay was performed and (**B**–**C**) the migration and invasion of 95C and A549 cells transfected with miR-29c inhibitors were determined. (**D**) The evaluation of transfection efficiency of miR-29c inhibitors. *p < 0.05, **p < 0.01, ***p < 0.001, data represent the means ± s.d.

It has become increasingly clear that TGF-β is critical for tumor progression, including EMT. Thus, we determined the expression of the miRNA-29c in TGF-β1 stimulated 95C and A549 cells and found that miRNA-29c was decreased by TGF-β1 (Figure 2A). Moreover, the level of Sp1 was increased upon the treatment of TGF-β1 in two lung cancer cells (Figure 2A), suggesting that miR-29c might function as a tumor suppressor and impair the TGF-β1-induced EMT.

Transwell assay was performed to determine the function of miR-29c in the tumor metastasis. The 95C and A549 cells were ectopically expressed miR-29c inhibitors (Figure 2D). The results shown that overexpression of miR-29c inhibitors could enhance the ability of migration and invasion of 95C and A549 cells (Figure 2B and 2C), which indicated that the inhibition of miR-29c was conducive to the metastasis in lung cancer.

### MiR-29c represses the TGF-β1-induced EMT

**Figure 3:**
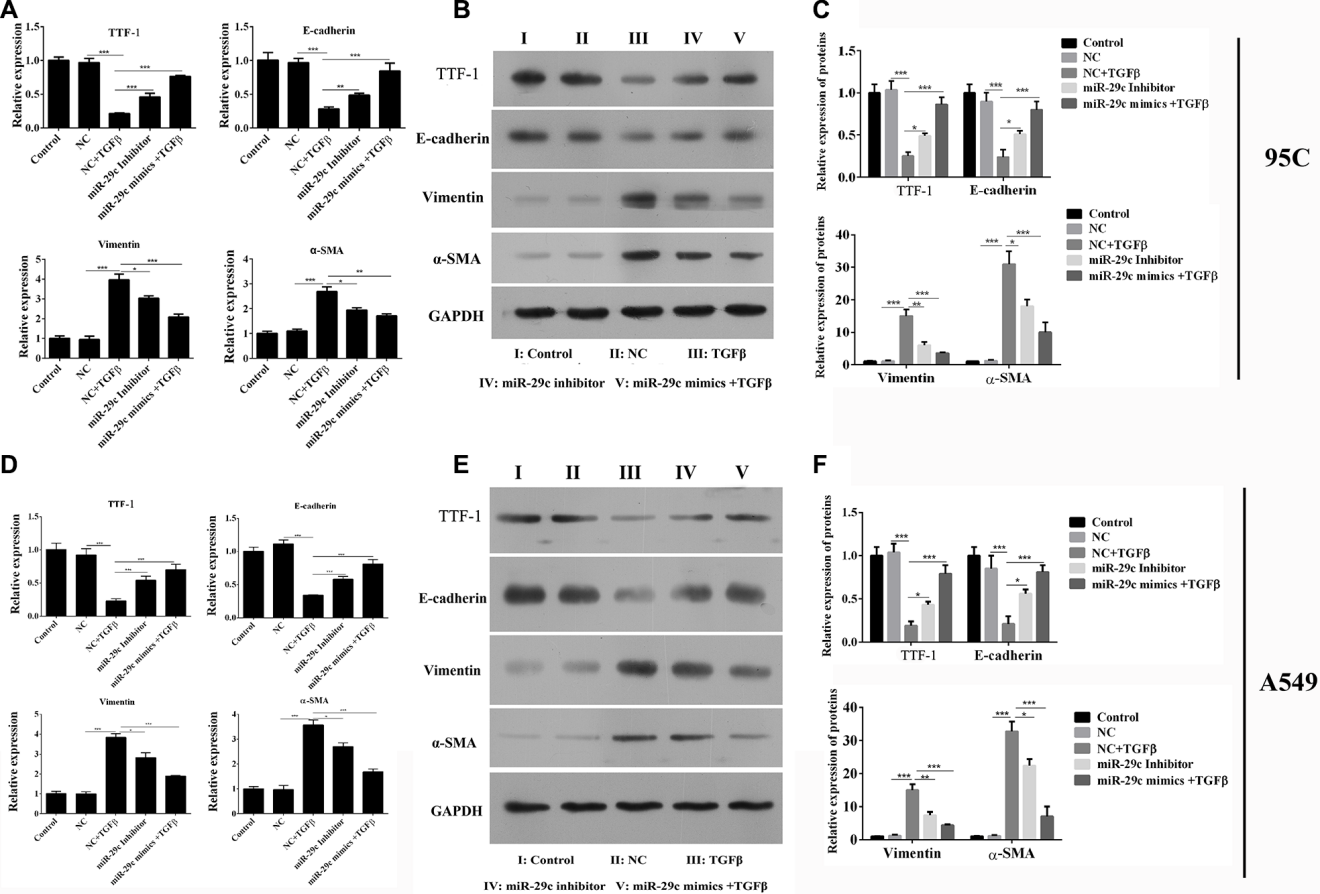
MiR-29c represses the TGF-β1-induced EMT The mRNA and protein expression of TGF-β1-induced EMT-associated markers including TTF, E-cadherin, vimentin and α-SMA were analyzed in (**A**–**C**) 95C and (**D**–**F**) A549cells with ectopically expressing miR-29c inhibitors or mimics. *p < 0.05, **p < 0.01, ***p < 0.001, data represent the means ± s.d.

We had demonstrated that the miR-29c could inhibit the migration and invasion of 95C and A549 cells, then the effects of miR-29c on EMT was determined. As previous studies reported, TGF-β1 induced the changes in EMT-associated markers in two cell lines, such as thyroid transcription factor 1 (TTF-1), E-cadherin, vimentin and α-smooth muscle actin (α-SMA). But we found that ectopically expressing miR-29c inhibitors without the treatment of TGF-β1 inhibited the mRNA expression of TTF-1 and E-cadherin, and enhanced the expression of vimentin and α-SMA, which indicated that the inhibition of miR-29c could be sufficient to induce EMT in lung cancer. In addition, we overexpressed the miR-29c mimics into 95C cells with the treatment of TGF-β1. The mRNA of EMT-associated markers were analyzed and we found that overexpression of miR-29c significantly repressed the TGF-β1-induced down-regulation of TTF-1 and E-cadherin, and up-regulation of vimentin and α-SMA (Figure 3A and 3D). The protein levels of EMT-associated markers were also confirmed by western blotting which shown the similar results, indicating that miR-29c could reverse the TGF-β1-induced EMT in 95C cell line (Figure 3B and 3C) and A549 cell line (Figure 3E and 3F).

### Sp1 overexpression could restore the miR-29c-induced inhibition of EMT

**Figure 4:**
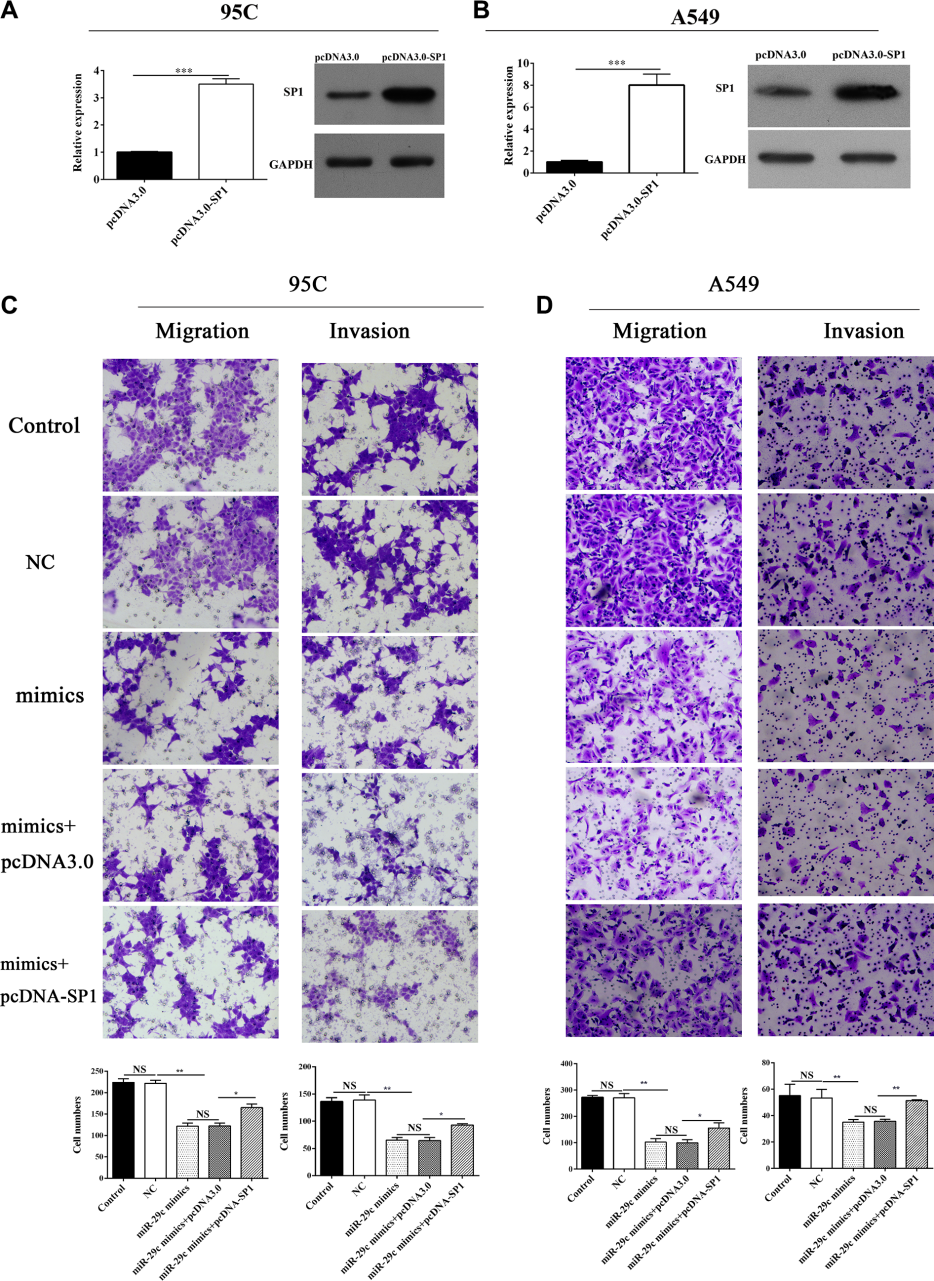
Sp1 could restore the miR-29c-induced inhibition of metastasis (**A**–**B**) Overexpression of Sp1 by transfection of pcDNA-Sp1 into 95C and A549 cells. Transwell assay was performed and (**C**–**D**) the migration and invasion of 95C and A549 cells transfected with miR-29c mimics or pcDNA-Sp1 were determined.

Sp1 had been found to be directly inhibited by miR-29c in lung cancer, and then we next investigated its role in tumor metastasis. We conducted the over-expression plasmid of Sp1 (Figure 4A and 4B), and transwell assay was performed. 95C cell and A549 cells were ectopically expressed of miR-29c mimics and Sp1 could remarkably enhance the ability of migration and invasion (Figure 4C and 4D) which indicated that the overexpression of Sp1 could abrogate the miR-29c-induced inhibition of metastasis in lung cancer.

**Figure 5:**
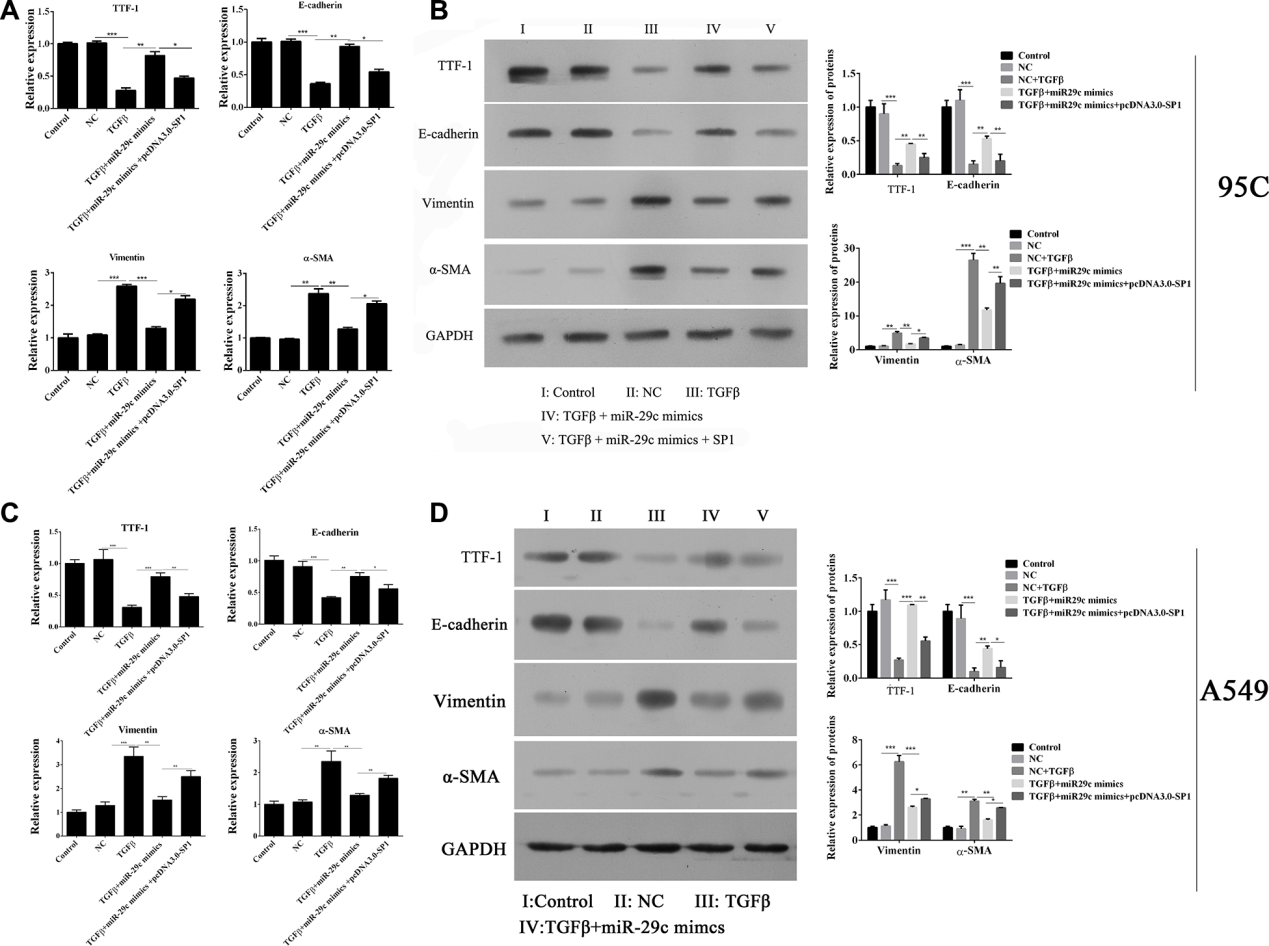
Overexpression of Sp1 impairs miR-29c-induced inhibition of EMT The mRNA and protein expression of TGF-β1-induced EMT-associated markers including TTF, E-cadherin, vimentin and α-SMA were analyzed in (**A**–**B**) 95C and (**C**–**D**) A549 cells with ectopically expressing miR-29c mimics or pcDNA-Sp1. *p < 0.05, **p < 0.01, ***p < 0.001, data represent the means ± s.d.

We had confirmed that overexpressed the miR-29c mimics into 95C cells and A549 cells were able to significantly impair the TGF-β1-induced EMT. Thus, the role of Sp1 in EMT was analyzed and the results indicated that overexpression of Sp1 in 95C cells could abrogate the miR-29c-induced inhibition of EMT by the up-regulation of TTF-1 and E-cadherin, and down-regulation of vimentin and α-SMA in mRNA (Figure 5A and 5C) and protein level (Figure 5B and 5D).

### miR-29c inhibits TGF-β1/Sp1 network and tumor progression *in vivo*

**Figure 6:**
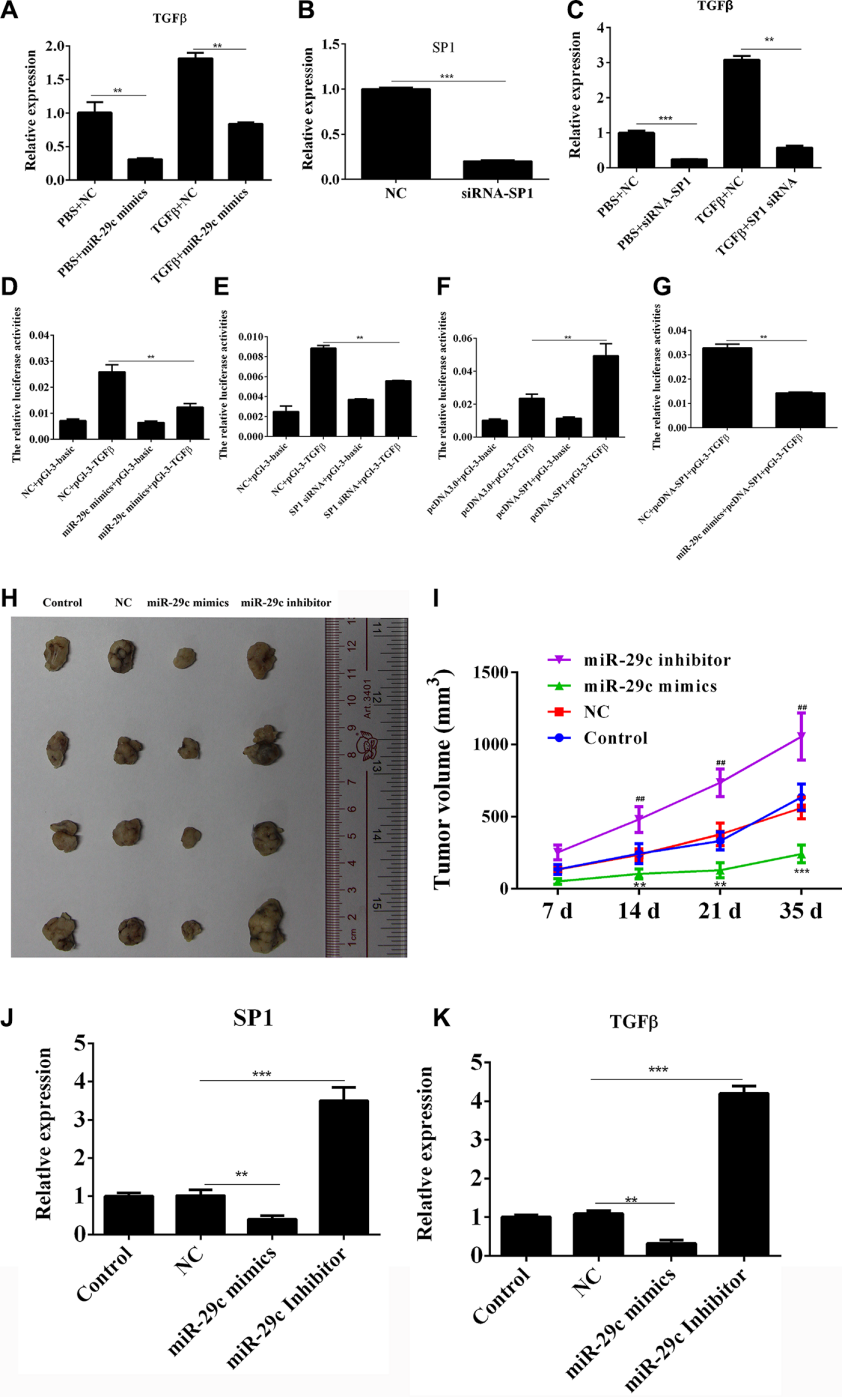
MiR-29c could inhibit Sp1/TGF-β1 expression and lung cancer progression (**A**) The TGF-β1 expression in overexpression of miR-29c mimics or negative control was analyzed by Q-PCR in 95C cells with or without the treatment of TGF-β1. (**B**–**C**) The TGF-β1 expression in overexpression of siRNA-Sp1or negative control was analyzed by Q-PCR in 95C cells with or without the treatment of TGF-β1. (**D**) The relative luciferase activities in 95C cells were determined after the pGL-3-TGFB1 plasmids were transfected with miR-29c mimics. (**E**–**F**) The relative luciferase activities in 95C cells were determined after the pGL-3-TGFB1 plasmids were transfected with siRNA-Sp1 or pcDNA-Sp1. (**G**) The relative luciferase activities in 95C cells were determined after the pGL-3-TGFB1 plasmids were transfected with pcDNA-Sp1 and miR-29c mimics. (**H**–**I**) Nude mice (6 per group) were subcutaneously injected of 3 × 10^6^ A549 cells and the miR-29c mimics, inhibitor or negative control (10 nM per injection) were delivered via intra-tumoral injection for six times, three days apart. The tumor volume (mm^3^) and body weight (g) were measured. (**J**–**K**) The levelS of Sp1 and TGF-β1 in tumor tissues were estimated. *p < 0.05, **p < 0.01, ***p < 0.001, data represent the means ± s.d.

Using the gain- and loss-of-function approaches, we analyzed the regulation of miR-29c/Sp1 on the expression of the gene TGFB1 which encodes TGF-β1. On one hand, ectopically expressing miR-29c in 95C cells without the treatment of TGF-β1 could significantly inhibit the mRNA expression and overexpression of miR-29c in 95C cells with treatment of TGF-β1 was also able to down-regulate the TGF-β1 expression when compared to the 95C cell transfected with negative control miRNA (Figure 6A). On another hand, we found that ectopically expressing siRNA of Sp1 in 95C cells with or without the treatment of TGF-β1 could significantly inhibit the mRNA expression (Figure 6B and 6C).

We further investigated the transcriptional regulation of miR-29c and Sp1 on TGFB1 and luciferase reporter assay was performed. The results showed that miR-29c mimics and siRNA-Sp1 could significantly impair the luciferase activity of TGFB1 (Figure 6D and 6E), but overexpression of Sp1 could, in turn, enhance the transcription of TGFB1 (Figure 6F). Moreover, ectopically expressing miR-29c mimics in 95C cells could repress the Sp1-induced enhancement of TGFB1 transcription via directly targeting Sp1 (Figure 6G).

We next estimated the role of miR-29c in tumor progression *in vivo*. The miR-29c mimics, inhibitor or negative control were delivered via intra-tumoral injection for six times, three days apart. The mice treated with miR-29c mimics had decreased Sp1/ TGF-β1 expression in tumor tissue and showed a delayed tumor growth with less tumor volume than that treated with PBS or negative control (Figure 6H and 6I). Whereas, the treatment of miR-29c inhibitor promote the tumor progression with enhanced of Sp1/ TGF-β1 expression (Figure 6D and 6E). These data indicated that miR-29c could target Sp1/TGF-β1 loop to repress carcinogenesis of lung cancer *in vivo*.

## DISCUSSION

**Figure 7:**
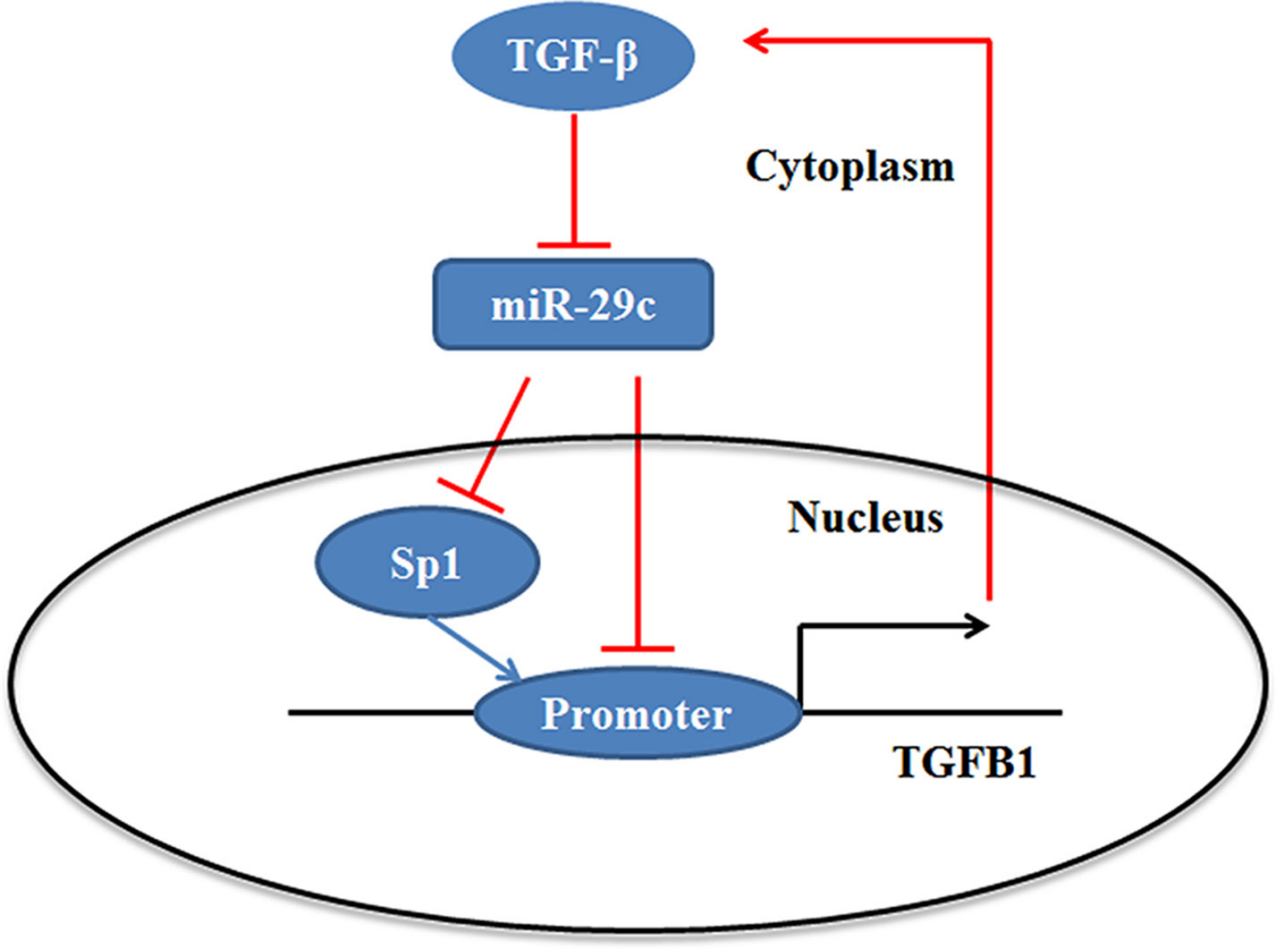
Summary diagram describes the miR-29c/Sp1 network that regulates TGF-β1 expression and TGF-β1-induced EMT

In this study, we investigated the potential mechanism of lung cancer metastasis and found that Sp1 could be inhibited by miR-29c in lung cancer and inhibition of miR-29c/ Sp1 dramatically enhanced the cell migration and invasion via the regulation of TGF-β-induced EMT. These results support the idea that TGF-β1 not only impaired the miR-29c/Sp1 axis, but could be inhibited by miR-29c, which leading to forming the miR-29c/Sp1/TGF-β1 network in lung cancer progression (Figure 7).

The Sp1-target genes involve into the hallmarks of cancer, which both activates and suppresses the expression of genes including prominent oncogenes and tumor suppressors, as well as genes involved in important biological processes in cell growth and differentiation [6]. Kerstin et al. had reported that Sp1 mediated through transcriptional induction of vimentin and cooperated with activated Smad complexes in mesenchymal transition and migration of pancreatic cancer cells for the TGF-β-induced EMT [16]. T-cell lymphoma invasion and metastasis 2 (TIAM2) could be positively controlled by Sp1 which was reported to promotes cell invasion and motility in NSCLC by activating EMT-associated genes [10]. We here found that, in lung cancer, Sp1 could form an auto-regulatory loop with TGF-β1, which enhanced TGFB1 transcription and TGF-β-induced EMT.

Although Sp1 was found to be targeted by miR-29c, this relationship in TGF-β-induced EMT in carcinogenesis is unknown. Xiao et al. revealed that miR-29c/Sp1 signals participated into the chemotherapy resistance to temozolomide (TMZ) in glioma [17]. Besides, in renal fibrosis, miR-29c/Sp1 signals could inhibit type I collagen production under TGF-β1-stimulated kidney fibrosis [15]. We here demonstrated that Sp1 could remarkably enhance the ability of migration and invasion of lung cancer, and miR-29c could directly target the Sp1-3’UTR and impair the TGF-β1-induced EMT via Sp1. Interestingly, zhang et al. had found that miR-29c could inhibit EMT and abrogate Wnt/β-catenin signaling pathway in human colorectal carcinoma. In addition, miR-29c impaired the CRC cells migration and invasion abilities *in vitro* and cancer metastasis *in vivo* by directly targeting guanine nucleotide binding protein alpha13 (GNA13) and protein tyrosine phosphatase type IVA (PTP4A) [14]. Additionally, miR-29c suppresses lung cancer cell adhesion to extracellular matrix (ECM) and metastasis by directly inhibiting integrin β1 and MMP2 expression and by further reducing MMP2 enzyme activity [18]. Thus, miR-29c could serves as a tumor metastasis suppressor, which negatively controls the cancer metastasis via targeting many molecules including Sp1, GNA13, PTP4A, integrin β1 and MMP2.

In breast cancer, miR-22 could indirectly inhibit CD147-associated tumor invasion and metastasis by repressing Sp1 expression, and form an auto-regulatory loop with Sp1 by binding to the miR-22 promoter to inhibit miR-22 transcription [19]. In this study, we found that TGF-β1 could inhibit the expression of miR-29c, which up-regulate the Sp1 expression for the induction of EMT. Meanwhile, TGF-β1 could be restored by decreased miR-29c/Sp1 signals, leading to enhanced TGF-β1 expression.

Our study provides the evidence that, in lung cancer, Sp1 could be repressed by miR-29c in translational level. TGF-β1 could inhibit the miR-29c/Sp1 axis, and in turn, the impaired miR-29c/Sp1 axis formed an auto-regulatory loop with TGF-β1, which enhances TGFB1 transcription for TGF-β1-induced EMT.

## MATERIALS AND METHODS

### Tumor samples, cell lines and reagents

A total of 20 lung cancer samples including tumor (T) and paracancerous tissues (N) were collected. According to WHO classifications, all the slides of lung cancer tissues were evaluated by two pathologists. This study was approved by the Research Ethics Committee of Chongqing cancer Hospital & Institute & Cancer center. All these retrospective specimens were handled and made anonymous according to the ethical and legal standards. Written informed consent was obtained from all of the patients. This information was supplied in the revised manuscript.

The paired low-metastatic 95C and high-metastatic 95D cell lines were subcloned from a low differentiated human large cell lung carcinoma cell line PLA-801 which were well authenticated and published by several research groups [20, 21]. The 95C, 95D cells were cultured in RPMI 1640 (Invitrogen, USA) with 100 units/mL penicillin, 100 μg/mL streptomycin and 10% calf bovine serum, and grown at 37°C in atmosphere with 5% CO_2_. The A549 and BEAS-2B cell lines were cultured in Dulbecco’s Modified Eagle’s medium (DMEM) supplemented with 10% FBS (Life Technologies, USA), ampicillin and streptomycin at 37°C, 5% CO_2_ conditions. Oligonucleotide sequences of miR-29c mimics, inhibitor, siRNA-Sp1 was purchased from RiboBio (Guangzhou, china). Reporter plasmid of full-length 3′-UTR (wild-type or mutant) of Sp1 mRNA and pGL-3-TGFB1 was conducted by GenePharma (Shanghai, China). For expression of Sp1, the overexpression vector pcDNA3.0-Sp1 was conducted by GenePharma (Shanghai, China). All constructs were finally confirmed by sequencing. E-Cadherin, TIF-1, vimentin, a-SMA and sp1 antibodies were obtained from Cell Signaling Tech (Denver, MA).

### Cell transfection

The 95C and A549 cells were cultured to about 80% confluence in 6-well plates and were transfected with Lipofectamine 2000 (Invitrogen, USA) according to the manufacturer’s instructions. After transfection for the indicated time, the cells were harvested for further experiments.

### Cell migration and invasion assays

The Transwell assay was performed to evaluate the potential for migration and invasion of transfected cells. In the migration assay, 2.5 × 10^4^ cells were cultured in the upper chamber of a non-coated transwell insert. While in the lower chamber, 600 μl medium with 10% bovine serum was used as a chemo-attractant to encourage cell migration. In the invasion assay, the upper chamber of the transwell inserts were coated with 50 μl of 2.0 mg/ml Matrigel, in the same time 5 × 10^4^ cells were plated in the upper chamber of the Matrigel-coated transwell insert. Cells were incubated for 24 h and cells that did not migrate or invade were removed using a cotton swab. The cells were stained using crystal violet staining and counted under an inverted microscope. Four random views were selected to count the cells and the independent experiments were repeated three times.

### Real-time PCR

According to the standard protocol, quantitative real-time RT-PCR (qRT-PCR) was performed, and the expression levels of Sp1 were normalized to GAPDH for gene expression. The primers were listed in Table 1.

**Table 1:** Primers for qPCR

Gene	Primer(5′-3′)
miR-29c-3p-F	ACACTCCAGCTGGGTAGCACCATTTGA
miR-29c-3p-R	TGGTGTCGTGGAGTCG
U6 F	CTCGCTTCGGCAGCACA
U6 R	AACGCTTCACGAATTTGCGT
GAPDH-F	ACACCCACTCCTCCACCTTT
GAPDH-R	TTACTCCTTGGAGGCCATGT
E-cadherin-F	ATGGCTTCCCTCTTTCATCTC
E-cadherin-R	ATAGTTCCGCTCTGTCTTTGG
TTF-1-F	CTCCCCAGGAGTTAAAAGAG
TTF-1-R	CATAGTAGATACGCCGACCA
vimentin-F	CCTTGAACGCAAAGTGGAATC
vimentin-R	CGTGAGGTCAGGCTTGGAAAC
α-SMA-F	CCCTTGAGAAGAGTTACGAGTTG
α-SMA-R	TGATGCTGTTGTAGGTGGTTTC
TGFB1-F	AGCAACAATTCCTGGCGATAC
TGFB1-R	CAACCACTGCCGCACAACT

### Western blotting

The total proteins were extracted from 95C and A549 cells. 20–30 μg of total proteins were dissolved in SDS-PAGE loading buffer, separated on 4%–15% polyacrylamide gel, transferred to nitrocellulose membranes (Amersham Biosciences). The membranes were blocked in 5% non-fat milk in TBST buffer (Tris Buffer Saline containing 0.1% Tween-20) for 1 h at room temperature, and subsequently incubated overnight at 4°C by the appropriately diluted primary antibodies. After washing with TBST buffer, the blots were then incubated with HRP-conjugated secondary antibody for 1 h at room temperature. After washing with TBST buffer, the signal of proteins was detected.

### Dual-luciferase assay

The 95C cells were co-transfected containing 200 ng firefly Luciferase vector, 40 ng Renilla luciferase pRL-TK vector (Promega, USA) and pcDNA-Sp1 or siRNA-Sp or miR-29c. Firefly luciferase acted as a reporter gene and Renilla luciferase as a normalized control. The transfected cells were incubated for 48 h. Luciferase activity was measured using the Dual-Luciferase Reporter Assay System (Promega, USA).

### Tumor model

For investigate the tumor suppressive role of miR-29c *in vivo*, 3 × 10^6^ A549 cells were subcutaneously injected in rear flank of nude mice (6 per group). The miR-29c mimics, inhibitor or negative control (10 nM per injection) were purchased from RiboBio (Guangzhou, china) and were delivered via intra-tumoral injection for six times, three days apart.

### Statistical analyses

The statistical analyses were performed by using the Statistical Package for Social Sciences version 16.0 (SPSS 16.0, SPSS Inc., Chicago, IL, USA) and the Prism statistical software package (Version 5.0, Graphpad Software Inc.). Unpaired *t-test*s or Mann–Whitney *U* tests were used to compare the two groups, and multiple group comparisons were analyzed with one-way ANOVA. *P* < 0.05 was considered statistically significant. All experiments were performed at least three times.
